# Identification of Optimal Movement Patterns for Energy Pumping

**DOI:** 10.3390/sports11020031

**Published:** 2023-01-30

**Authors:** Micha Luginbühl, Micah Gross, Silvio Lorenzetti, David Graf, Martin J. Bünner

**Affiliations:** 1 Institute of Computational Engineering, University of Applied Sciences OST, Werdenbergstr. 4, CH-9470 Buchs, Switzerland; 2 Swiss Federal Institute of Sport (SFISM), CH-2532 Magglingen, Switzerland; 3 Institute of Biomechanics, D-HEST, ETHZ, CH-8092 Zurich, Switzerland; 4 Swiss Cycling, Sportstrasse 44, CH-5240 Grenchen, Switzerland

**Keywords:** energy pumping, ski-cross, BMX-racing, optimal control theory, SQP-algorithm, center of mass movement, rollers, digital technology, sports

## Abstract

Energy pumping is a way to gain kinetic energy based on an active vertical center of mass movement in rollers in sports like skateboarding, skicross, snowboard cross and BMX. While the principle of the energy transfer from the vertical movement to the horizontal movement is well understood, the question of how to achieve the optimal energy transfer is still unresolved. In this paper, we introduce an inverse pendulum model to describe the movement of the center of mass of an athlete performing energy pumping. On this basis, the problem of identifying the optimal movement pattern is formulated as an optimal control problem. We solve the discretized optimal control problem with the help of a SQP-algorithm. We uncover that the optimal movement pattern consists of a jumping, flying, and landing phase, which has to be timed precisely. We investigate how the maximal horizontal speed depends on parameters like rollers height and maximal normal force of the athlete. Additionally, we present a qualitative comparison of our results with measured results from BMX-racing. For athletes and coaches, we advice on the basis of our results that athlete’s performance is optimized by using maximal force and adopt an exact and proper timing of the movement pattern.

## 1. Introduction

In many sports like skateboarding, BMX, ski/snowboard cross, the racing track includes wave wells, moguls or rollers. The aim for the athlete is to loose as little time as possible and exit the rollers with a high speed. Here, the potential energy as well as specific movements of the center of mass (energy pumping) can be used to gain speed. For BMX racing the surprisingly high importance of energy pumping has been demonstrated in experiments [1].

Here we define energy pumping as all active and targeted movements of the center of mass of the athlete to generate kinetic energy in sports like skateboarding, skicross, snowboard cross, BMX and gymnastics. The energy transfer is common in sports eq. gymnastics [2] or sailing [3]. Also a swing can be pumped with vertical movements of the center of mass [4,5]. While descending a slope, the force in the direction of the slope can be used for acceleration and results in a larger speed. This accelerating force can be enlarged by a rise of the normal force during vertical acceleration of the center of mass.

In recent years, some progess has been made by formulating biomechanical problems as optimal control problems [6,7,8,9]. In this paper, the problem of identifying the optimal movement pattern for athlete’s energy pumping on a one-dimensional track is formulated as an optimal control problem, which is standard in the framework of optimal control theory [10]. The discretized optimal control problem turns out to be a high-dimensional nonlinear program (NLP). In this formulation, the athlete’s optimal movement pattern is a solution of the KKT-equations [11], derived from the NLP, in a high-dimensional real-valued space. The task to find these KKT-points in high-dimensional spaces is in practice extremely challenging. More precisely, it is far beyond what humans can do without help.

Since not so long ago, the SQP-algorithm, as proposed by Han, and Powell [12,13], and Schittkowski [14], has been shown to be able to identify KKT-points in the high-dimensional real-valued search space. In practice, the SQP-algorithm, running on a computer, can find KKT-points in spaces with several hundred or thousands dimensions. But, the effort to find these solutions is tremendous and without the help of computers, we were not able to find those. Also the optimal movement patterns, which will be presented in this paper, would not be accessible without the help of computers, which perform step-by-step intelligent numerical mathematics procedures. As such, the SQP-method is considered for good reasons as one tool out of the rich toolbox of artificial intelligence.

Historically, as the SQP-method was increasingly available to the scientific and engineering community and the 1990’s and 2000’s, it enlarged the set of problems, which can be solved. No wonder that the SQP-method was used in several engineering disciplines, like radio frequency design [15], wind turbine airfoil design [16], turbine design [17], mechanical engineering [18], and 2D airfoil design [19], to identify optimal designs, not known to engineers until then.

The optimal vertical movement during energy pumping including friction and different forms of moguls is not well understood. Therefore the aim of this work is to build a model based on the law of motions and find the optimal path.

## 2. The Inverted Pendulum Model and the Optimal Control Problem

### 2.1. The Inverted Pendulum Model

Inverted-pendulum models are proven tools as a simplified description of complex human movements [20,21]. To our knowledge, an inverted-pendulum-model for the energy pumping performed by an athlete has not been reported elsewhere. Therefore, we will present the model in detail in this section. We consider a one-dimensional track of length *L* with a height profile z=u(x),0≤x≤L. The athlete moves in positive x-direction. The local slope of the track is $u^{\prime }\left(x\right)$ and the angle between the *x*-axis and the track is $\phi \left(x\right)=\arctan(u^{\prime }\left(x\right))$. The vector $\vec{N}$ is the upwards-pointing normal vector on the track u(x):

(1) $$\vec{N}\left(x\right)=\left(\;-\sin\left(\phi \left(x\right)\right),\;\cos\left(\phi \left(x\right)\right)\;\right),$$

which we will call *track normal unit vector* in the following.

**Figure 1:**
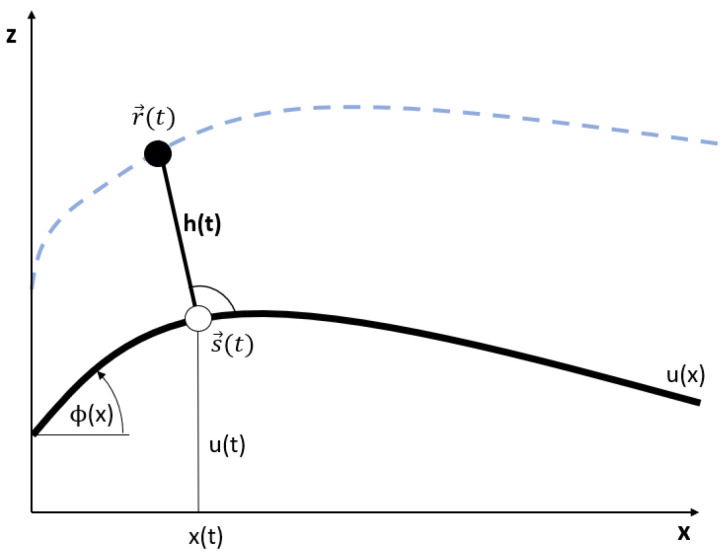
Inverted pendulum model for athlete’s movement along the track with height profile u(x) (thick line). Dashed line: Trajectory of the COM (full circle). The contact point is shown as an open circle.

We model the athlete, including his equipment, as an inverted pendulum, moving from x=0 to x=L:

1. The athlete, including his or her equipment, has a single contact point
  (2) $$\vec{s}\left(t\right)=\left(\;x\left(t\right),\;u\left(x\left(t\right)\right)\;\right),$$
  at which he is in contact with the track for all times (see Figure 1). From now on, we will write athlete only, instead of athlete, including his or her equipment for simplicity. The athlete’s mass is concentrated in the center of mass (COM) located at
  (3) $$\vec{r}\left(t\right)=\left(\;r_{x}\left(t\right),\;r_{z}\left(t\right)\;\right).$$
2. The body axis of the athlete is modelled as a straight line between the contact point positioned at $\vec{s}\left(t\right)$ and the COM positioned at $\vec{r}\left(t\right)$. The straight line is considered as a stiff bar in the sense that forces can be transferred to the COM from the contact point along the straight line.
3. The body axis is always perpendicular to the track. Therefore, the vector $\vec{h}\left(t\right)=\vec{s}\left(t\right)-\vec{r}\left(t\right)$ is perpendicular to the track, and parallel to $\vec{N}\left(x\left(t\right)\right)$. The length of $\vec{h}\left(t\right)$ we call effective body height: $h\left(t\right)=|\vec{h}\left(t\right)|$. With this, the location of the COM can be computed from the location of the contact point and the effective body height: $\vec{r}\left(t\right)=\vec{s}\left(t\right)+h\left(t\right)\cdot N\left(x\left(t\right)\right)$. As the athlete bends and stretches his body, he or she can vary his or her effective body height between the minimum effective body height $h_{min}$ (body bent maximally) to the maximal effective body height $h_{max}$ (body stretched maximally).
4. As the athlete moves along the track, a normal force acts from the track to the athlete’s COM. This force acts along the body axis, from the contact point to the COM. Therefore, the athlete in our model has to make sure that his or her body axis is exactly parallel to the normal forces. This is in contrast to some sports like cycling, or skiing, there the athlete can balance forces by leaning backward or forward, since it equipment (ski, bike) provides an extended contact line, or several isolated contact points, between the athlete and the track.

With this, we arrive at a 2-dimensional dynamical problem for the movement of the COM with:

(4) $$\vec{v}=\dot{\vec{r}},\;\vec{a}=\ddot{\vec{r}},\;v=|\vec{v}\left|,\;a=\right|\vec{a}|.$$

Additionally, we introduce (a) the trajectory tangential unit vector $\vec{T}\left(\dot{\vec{r}}\right)$, which is tangential to the movement of the COM for all times, and (b) the trajectory normal unit vector $\vec{R}\left(\dot{\vec{r}},\ddot{\vec{r}}\right)$, which points from the COM to the middle of the curvature circle.

(5) $$\begin{array}{ccc} \vec{T}\left(\dot{\vec{r}}\right) & = & \frac{\vec{v}}{v}\;, \end{array}$$

(6) $$\begin{array}{ccc} \vec{R}\left(\dot{\vec{r}},\ddot{\vec{r}}\right) & = & \frac{\dot{\vec{T}}}{|\dot{\vec{T}}|}\;,\;\left|\dot{\vec{T}}\right|\neq 0. \end{array}$$

The curvature of the COM’s path, defined as the inverse of the curvature radius, is:

(7) $$k\left(\dot{\vec{r}},\ddot{\vec{r}}\right)=\frac{|\vec{v}\times \vec{a}|}{v^{3}}$$

**Figure 2:**
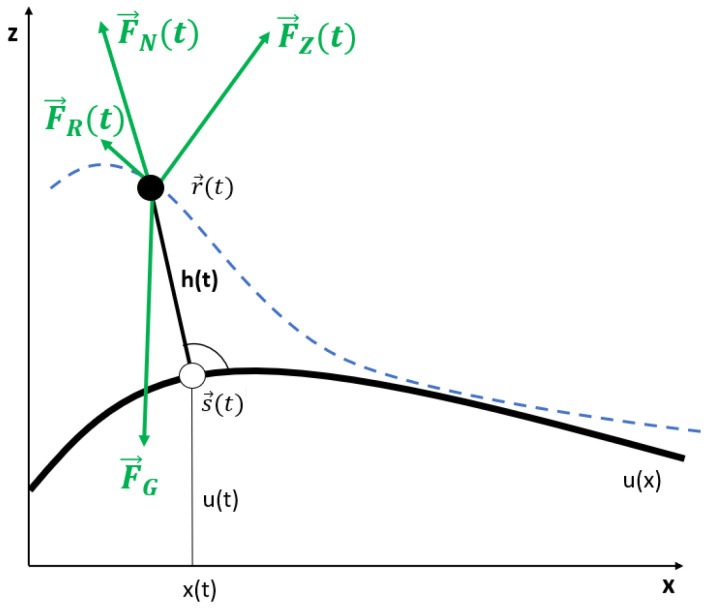
The movement of the COM (full circle) along his continuous path (dashed line) of the movement of the COM (full circle). The contact point is shown as an open circle.

Now, let us consider the forces, respectively accelerations, acting on the athlete’s COM. These are gravity, friction, body force, and centrifugal forces. The gravity force, respectively acceleration is (see Figure 2):

(8) $${\vec{F}}_{G}=\left(\begin{array}{c} 0\\ -mg \end{array}\right),\;{\vec{a}}_{G}=\left(\begin{array}{c} 0\\ -g \end{array}\right)$$

The centrifugal force, respectively acceleration is given by:

(9) $${\vec{F}}_{Z}\left(\dot{\vec{r}},\ddot{\vec{r}}\right)=mv^{2}k\left(\dot{\vec{r}},\ddot{\vec{r}}\right)\vec{R}\left(\dot{\vec{r}},\ddot{\vec{r}}\right),\;{\vec{a}}_{Z}\left(\dot{\vec{r}},\ddot{\vec{r}}\right)=v^{2}k\left(\dot{\vec{r}},\ddot{\vec{r}}\right)\vec{R}\left(\dot{\vec{r}},\ddot{\vec{r}}\right).$$

By stretching or folding his or her body along the body axis, the athlete is able to apply an additional force to the movement of the COM, which we call body force from now on. In the model presented, the athlete can only provide forces parallel to his or her body axis. Hence, the body force acts in the direction of the track normal unit vector $\vec{N}$.

(10) $${\vec{F}}_{N}\left(t,\vec{r}\right)=F_{N}\left(t\right)\cdot \vec{N}\left(\vec{r}\right)=m\cdot a_{N}\left(t\right)\cdot \vec{N}\left(\vec{r}\right).$$

For any time, we assume: (a) the body acceleration $a_{N}\left(t\right)$ cannot be negative, and (b) the body acceleration cannot be larger than a maximal value $a_{N}^{max}$, since the physical strength of the athlete is finite. Therefore, the restriction: $0\leq a_{N}\left(t\right)\leq a_{N}^{max}$ holds for all times.

In the model we introduce two friction forces, the air friction and the sliding or rolling friction:

(11) $${\vec{F}}_{R}\left(t,\vec{r},\dot{\vec{r}}\right)=-\left[\mu F_{N}\left(t,\vec{r}\right)+m\alpha \left(\vec{r},\dot{\vec{r}}\right)\right]\vec{T}\left(\dot{\vec{r}}\right).$$

The air friction is modelled with a simple Newtonian ansatz leading to frictional force with a quadratic law in *v*:

(12) $$\alpha \left(\vec{r},\dot{\vec{r}}\right)=\frac{c_{w}\rho A\left(\vec{r}\right)v^{2}}{2m},$$

with $c_{w}$ athlete’s $c_{w}$-value, ρ density of the medium (in our case: air), and a time-dependant cross-sectional area A(t) of the athlete. We model the variation of the cross-sectional area with the effective body height with the help of a simple linear law:

(13) $$A\left(\vec{r}\right)=\frac{A_{max}}{h_{max}}\cdot h\left(\vec{r}\right),$$

with $A_{max}$ being the athlete’s cross-sectional with a fully stretched body. The sliding or rolling friction is modelled to be proportional to the normal force with the coefficient μ.

We are now in the position to establish the equations of motion for the movement of the COM with the help of Newton’s law:

(14) $$m\ddot{\vec{r}}={\vec{F}}_{R}\left(t,\vec{r},\dot{\vec{r}}\right)+{\vec{F}}_{G}+{\vec{F}}_{N}\left(t,\vec{r}\right)+{\vec{F}}_{Z}\left(\dot{\vec{r}},\ddot{\vec{r}}\right),$$

As a result we can derive an implicit non-autonomous set of second order differential equations for the movement of the COM, by applying the expressions for the accelerations as derived earlier in this paper:

(15) $$\ddot{\vec{r}}=-\left[\mu a_{N}\left(t\right)+\alpha \left(\vec{r},\dot{\vec{r}}\right)\right]\vec{T}\left(\dot{\vec{r}}\right)+v^{2}k\left(\dot{\vec{r}},\ddot{\vec{r}}\right)\vec{R}\left(\dot{\vec{r}},\ddot{\vec{r}}\right)+a_{N}\left(t\right)\cdot \vec{N}\left(\vec{r}\right)+{\vec{a}}_{G},$$

with either:

1. initial conditions:
  (16) $$\dot{\vec{r}}\left(0\right)={\vec{v}}_{0},\;\vec{r}\left(0\right)={\vec{r}}_{0},$$
2. or circular conditions:
  (17) $$\dot{\vec{r}}\left(0\right)=\dot{\vec{r}}\left(\tau \right),\;\vec{r}\left(0\right)=\vec{r}\left(\tau \right).$$

With circular conditions (17), we model a situation in which the athlete moves on a periodic track, where the shape of the track repeats itself over-and-over again (with infinite length). With initial conditions (16), we model a situation in which the athlete moves on a track with finite length *L* only once.

Note the implicitness of the set of differential Equation (15), since $\ddot{\vec{r}}$ appears on the left-hand side and the right-hand side of the equations. Therefore, straightforward ODE-solvers like Runge-Kutta-schemes, implementing a discretization in time, cannot be used for the numerical solution. As we will solve (15) as constraints to an optimization problem (to be shown later in this paper), the implicit nature of (15) is taken care of in a straightforward way.

We are interested in the solution of the set of ODEs for 0≤t≤τ with x(τ)=L. For initial conditions, τ is the time to complete the track of length *L*. For circular conditions, τ is the time to complete a single period of length *L* of the track (which has infinite length).

Note that the body acceleration $a_{N}\left(t\right)$ acts as an external driver, or external force, on the ODE-system (15) and needs to be fixed prior to the solution. As such COM’s trajectory $\vec{r}=\vec{r}\left[a_{N}\right]$ is a functional of $a_{N}\left(t\right)$. The same is true for the completion time $\tau =\tau [a_{N}]$.

It is important to note, that not all set of parameters, initial conditions, and body accelerations $a_{N}\left(t\right)$ lead to solutions, which satisfiy the implicit constraint $h_{min}\leq h\left(t\right)\leq h_{max}$. Therefore, we restrict the set of parameters, initial conditions, and body accelerations $a_{N}\left(t\right)$ to those who give rise to solutions which satisfy the implicit constraint. $h_{min}\leq h\left(t\right)\leq h_{max}$.

### 2.2. The Optimal Control Problem

The aim of this paper is to identify the optimal movement pattern for energy pumping under variation of the relevant parameters such aus athlete’s mass, friction or the shape of the track. The optimal movement pattern is defined as the one, which results in the fastest completion of the track, or, which gives the smallest overall time for track completion τ.

The athlete’s degree of freedom to modify the movement pattern is the acceleration $a_{N}\left(t\right)$, which encompasses the body movement of the athlete, including the variation of athlete’s cross sectional area $A\left(t\right)=A\left[a_{N}\right]\left(t\right)$, since the latter is also a functional of $a_{N}\left(t\right)$. This leads to a time-continuous optimal control problem with the function $a_{N}\left(t\right)$ as functional design variable:

(18) $$\begin{array}{ccc} \tau^{*} & = & \min_{a_{N}\left(t\right)}\tau \left[a_{N}\right], \end{array}$$

(19) $$\begin{array}{ccc} & h_{min} & \leq h\left[a_{N}\right]\left(t\right)\leq h_{max},\;0\leq t\leq \tau , \end{array}$$

(20) $$\begin{array}{ccc} & 0 & \leq a_{N}\left(t\right)\leq a_{N}^{max},\;0\leq t\leq \tau , \end{array}$$

(21) $$\begin{array}{ccc} & \ddot{\vec{r}} & =-\left[\mu a_{N}\left(t\right)+\alpha \left(\vec{r},\dot{\vec{r}}\right)\right]\vec{T}\left(\dot{\vec{r}}\right)+v^{2}k\left(\dot{\vec{r}},\ddot{\vec{r}}\right)\vec{R}\left(\dot{\vec{r}},\ddot{\vec{r}}\right)+a_{N}\left(t\right)\cdot \vec{N}\left(\vec{r}\right)+{\vec{a}}_{G}, \end{array}$$

(22) $$\begin{array}{ccc} & x(\tau ) & =L \end{array}$$

Additionally, we apply either initial value conditions (16) or circular conditions (17) to the ODE.

In less mathematical terms, the meaning of the optimization problem (18)–(22) is: Find the time-dependant body acceleration $a_{N}\left(t\right)$ such that the track u(x) is completed as fast as possible starting from x=0 to x=L, taking into account all restrictions imposed on the problem. The solution of the optimization problem delivers the optimal acceleration $a_{N}^{*}\left(t\right)$, the minimal possible completion time $\tau^{*}$, as well as the optimal trajectory of the COM of the athlete $\left({\dot{\vec{r}}}^{*},{\vec{r}}^{*}\right)$. All three together, forces and trajectory, defines the optimal movement pattern of the athlete.

**Figure 3:**
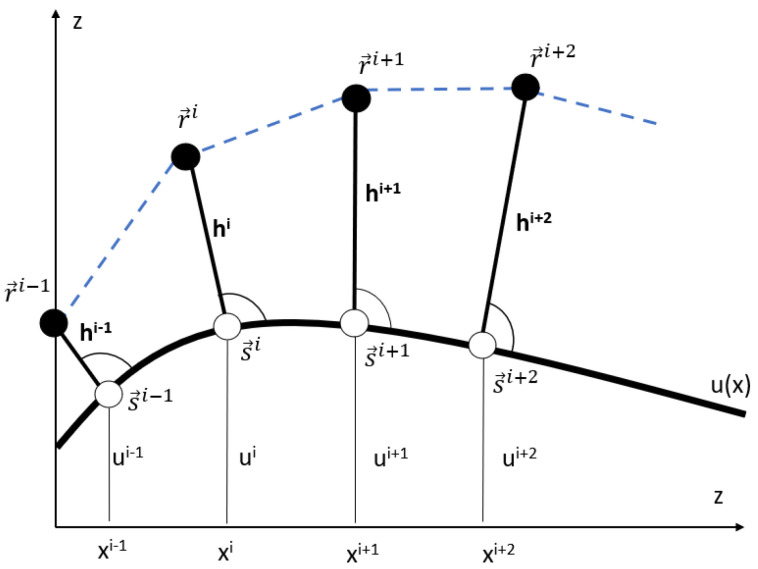
Polygonal line (dashed line) of the movement of the COM (full circle). The base points are shown as open circles.

In order to solve the optimal control problem, we discretize the model in space. To this end, we introduce the equidistant discretization of the x-coordinate of the contact point with an interval Δx: $x_{i}^{s}=\left(i-1\right)\Delta x,i=1,\ldots N$ with $N=\frac{L}{\Delta x}+1$. The track is discretized as ${\vec{s}}_{i}=\left(x_{i}^{s},\;u\left(x_{i}^{s}\right)\right)$, also $\phi_{i}=\arctan\left(u^{\prime }\left(x_{i}^{s}\right)\right)$ and the discretized normal vector is ${\vec{N}}_{i}=\left(-\sin\left(\phi_{i}\right),\;\cos\left(\phi_{i}\right)\right)$. Accordingly, discretizations apply as: $h_{i}=h\left(x_{i}^{s}\right)$ und ${\vec{r}}_{i}={\vec{s}}_{i}+h_{i}\cdot {\vec{N}}_{i}$. As such, the continuous movement of the COM $\vec{r}\left(t\right)$ is approximated by an polygonal line (see Figure 3).

On this basis, we define (3N−1) design variables $\left(h^{i},v^{i},a_{N}^{i}\right)$ for the time-discrete optimal control problem, with $h^{i}=\left(h^{1},\ldots ,h^{N}\right),v^{i}=\left(v^{1},\ldots ,v^{N-1}\right),a_{N}^{i}=\left(a_{N}^{1},\ldots ,a_{N}^{N}\right)$. Here, $v^{i}$ is the magnitude of the velocity as the COM moves from ${\vec{r}}^{i}$ to ${\vec{r}}^{i+1}$. From the design variables together with the parameters all dynamical variables can be computed. The discretized version of accelerations $\left(a_{x}^{i},a_{z}^{i}\right)$ are computed with the help of first-order finite differences from the velocities. With this, the discretized versions of the tangential and radial vectors are computed. The time for the COM to move from *i* to (i+1) is:

(23) $$\Delta \tau^{i}=\frac{|{\vec{r}}^{i+1}-{\vec{r}}^{i}|}{v^{i}}$$

The overall time τ with x(τ)=L, which is needed to complete the track, can be computed as a sum of N−1 time steps:

(24) $$\tau \left(h^{i},v^{i}\right)=\sum \Delta \tau^{i}=\sum \frac{|{\vec{r}}^{i+1}-{\vec{r}}^{i}|}{v^{i}}$$

As a result, the optimization problem reads:

(25) $$\begin{array}{ccc} \tau^{*} & = & \min_{\left(h^{i},v^{i},a_{N}^{i}\right)}\tau \left(h^{i},v^{i}\right), \end{array}$$

(26) $$\begin{array}{ccc} & h_{min} & \leq h^{i}\leq h_{max},\;i=1,\ldots N \end{array}$$

(27) $$\begin{array}{ccc} & a_{N}^{min} & \leq a_{N}^{i}\leq a_{N}^{max},\;i=1,\ldots N \end{array}$$

(28) $$\begin{array}{ccc} & v^{i} & \geq 0,\;i=1,\ldots N-1 \end{array}$$

(29) $$\begin{array}{ccc} & a_{x}^{i} & =-\left[\mu a_{N}^{i}+\alpha \left(\vec{r^{i}},\dot{\vec{r^{i}}}\right)\right]{\vec{T}}_{x}\left(\dot{\vec{r^{i}}}\right)+{\left(v^{i}\right)}^{2}k\left(\dot{\vec{r^{i}}},\ddot{\vec{r^{i}}}\right){\vec{R}}_{x}\left(\dot{\vec{r^{i}}},\ddot{\vec{r^{i}}}\right)+a_{N}^{i}\cdot {\vec{N}}_{x}\left(\vec{r^{i}}\right), \end{array}$$

(30) $$\begin{array}{ccc} & a_{z}^{i} & =-\left[\mu a_{N}^{i}+\alpha \left(\vec{r^{i}},\dot{\vec{r^{i}}}\right)\right]{\vec{T}}_{z}\left(\dot{\vec{r^{i}}}\right)+{\left(v^{i}\right)}^{2}k\left(\dot{\vec{r^{i}}},\ddot{\vec{r^{i}}}\right){\vec{R}}_{z}\left(\dot{\vec{r^{i}}},\ddot{\vec{r^{i}}}\right)+a_{N}^{i}\cdot {\vec{N}}_{z}\left(\vec{r^{i}}\right)+g, \end{array}$$

In case of simple initial conditions, we additionally have the constraints:

(31) $$\begin{array}{ccc} v^{1} & = & v_{0}, \end{array}$$

(32) $$\begin{array}{ccc} h^{1} & = & h_{0}. \end{array}$$

In case of circular initial conditions, we additionally have the constraints:

(33) $$\begin{array}{ccc} v^{1} & = & v^{N-1}, \end{array}$$

(34) $$\begin{array}{ccc} h^{1} & = & h^{N-1}, \end{array}$$

(35) $$\begin{array}{ccc} h^{2} & = & h^{N}. \end{array}$$

In case of simple initial conditions, the optimization problem is an NLP with 3N−1 real-valued design variables, 5N inequality constraints and 2N+2 equality constraints. In case of simple initial conditions, the optimization problem is an NLP with real-valued 3N−1 design variables, 6N inequality constraints and 2N+3 equality constraints.

## 3. Results

### 3.1. Solution of the Discrete Optimal Control Problem with a SQP-Algorithm

At first, we present the solution of the discretized optimal control problem (25)–(30) with the help of a SQP-algorithm as proposed by Han, and Powell [12,13]. We use circular conditions (17), with the length of a single period of the track L=6.0 m. The height profile is a simple cosinus-curve with a height difference of 0.2 m between highest and lowest point: $u\left(x\right)=(1-\cos\left(\frac{\pi x}{3}\right))\cdot 0.1$ m, and $\rho =1.225\frac{\mathrm{kg}}{m^{3}}$ For the athlete’s parameters we take: m=75 kg; $A^{max}=1.1$ m${}^{2};c_{w}=0.3;\mu =0.02;a_{N}^{min}=0;a_{N}^{max}=2g;h^{min}=0.7$ m; $h^{max}=1.2$ m with $g=9.81\frac{m}{s^{2}}$.

We choose an interval of Δx=0.1 m for the spatial discretization step, and therefore, N=61. On this basis, the NLP (25)–(30) is formulated with 182 design variables, 305 linear inequality constraints, and 124 nonlinear equality constraints. It is important for us to highlight the following: (a) with 182 design variables the search is performed in a 182-real-valued space, which is a tremendously large search space. (b) The 124 nonlinear equality constraints restrict the feasible region to a 62-dimensional subspace. Even if this sounds large, in fact, the feasible region is much, much smaller compared to the search space. (c) As a result, we expect it to be non-trivial to find the small feasible solution of the optimal control problem starting from an infeasible initial value.

Not surprisingly, it turned out, that random initial values for the search in the 182-dimensional space are in almost any case too far away from the feasible region, such that the SQP-algorithm was unable to find the feasible region, and therefore did not manage to solve the problem. Alternatively, we used random initial values of the form $u_{0}=\left(h_{0}\cdot \left(1,1,\ldots 1\right),v_{0}\cdot \left(1,1,\ldots 1\right),a_{N,0}\cdot \left(1,1,\ldots 1\right)\right)$ with random numbers $h_{0},v_{0}$, and $a_{N,0}$. With this, we solved the NLP with a set 1000 random initial conditions. From those 869 SQP-runs did not converge as the algorithm was unable to find the feasible region. And 131 SQP-runs converged to a local minimum, fulfilling the KKT-condition with high accuracy:

(36) $$|\nabla f+\Sigma \lambda_{i}\nabla g_{i}|\leq 10^{-6}.$$

**Figure 4:**
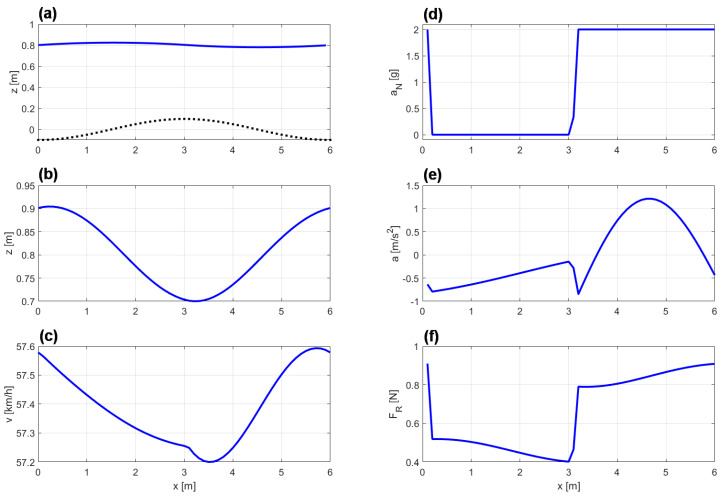
Optimal movement pattern, with $\tau^{*}=0.3770$ s, for a track of length L= 6.0 m and a sinusoidal height profile with a difference of 0.2 m between the lowest and the highest point: (**a**) track profile (dotted line), trajectory of the athlete’s COM (full line), (**b**) effective body height h(t), (**c**) magnitude of the velocity of the COM v(t), (**d**) magnitude of the normal acceleration $a_{N}\left(t\right)$, (**e**) magnitude of the acceleration of the COM a(t), and (**f**) magnitude of the total friction force $F_{R}\left(t\right)$.

In the subset of the converged SQP-runs, 96 times the algorithm converged to an identical local minimum with $\tau^{*}=0.3770$ s as presented in Figure 4. The remaining 35 times the algorithm converged to “exotic” local minima with unacceptable high values of the goal function: $\tau^{*}>3$ s. As such we can conclude that the optimal control problem ((25)–(30)) possesses several local minima, which is not surprising due to convex-concave nature of the problem. But the best local minima can be clearly identified as the “global” solution, and therefore we accept the local minimum presented in Figure 4 as the solution of ((25)–(30)).

Naturally, the equality constraints are all active, and therefore their corresponding Lagrange multipliers are positive. Beyond those, at the solution, in total 60 inequality constraints are active with positive Lagrange multipliers. From this, we can conclude, that we can accept the solution computed by the SQP-algorithm as the solution of the optimal control problem.

The calculations were done on a standard PC. We used the SQP-algorithm proposed by Han & Powell [12] as integrated in Matlab 2020a. Depending on the quality of the initial value for the SQP-algorithm, approximately 200 up to 1000 iterations were required to solve the optimal control problem with the required accuracy, which is a totally acceptable value for such a problem. Computation time on a standard time were approximately 1 min up to 10 min.

The athlete moves the COM in vertical direction with a minimal effective body height slightly after the track’s peak, and a maximal effective body height slightly after the track has reached its valley (Figure 4a,b). Note that the athlete does not stretch his or her body to the maximum, but only to a level slightly more than the track’s bump height. Quite obviously, it is advantageous for the athlete to keep the COM’s movement stable on the same height, while major movement is done by the legs, in accordance to what is observed in sports. The magnitude of athlete’s velocity varies between 57.2 km/h and 57.6 km, where the maximal velocity is reached slightly before the track’s valley and and minimal velocity is slightly after the track’s peak (Figure 4c).

In our eyes, the data presented in Figure 4d deliver a good insight into the optimal movement pattern, since the normal force $a_{N}\left(t\right)$ switches between 0 and 2g, which is the maximal acceleration to be applied by the athlete. As such, the optimal movement pattern can be understood as a jumping-flying-landing-motion. The jump is performed in such a way that the athlete leaves the track exactly at the valley and performs a parabola-flight over the hill. At the downhill-side of the hill, the athlete uses its maximal body force for the landing and the preparation for the next jump. Loosely speaking, the athlete avoids driving uphill by jumping. Instead, the athlete only drives downhill. This picture delivers an intuitive and yet scientifically correct understanding of the energy pumping on a one-dimensional track. In Figure 4f, we see that the friction force acting on the athlete is largely affected by the jumping and landing. In the flying-phase the friction force is considerably smaller than in the landing phase, since the there is no sliding or rolling friction in athlete’s flying-phase.

### 3.2. Optimal Movement Patterns under Variation of Athlete’s Maximal Body Force and Track Height

In this section, we investigate (a) the effect of maximal vertical body force, which the athlete can apply for energy pumping, and (b) the effect of the track height, on the horizontal velocity. The parameters are chosen as in the previous section.

**Figure 5:**
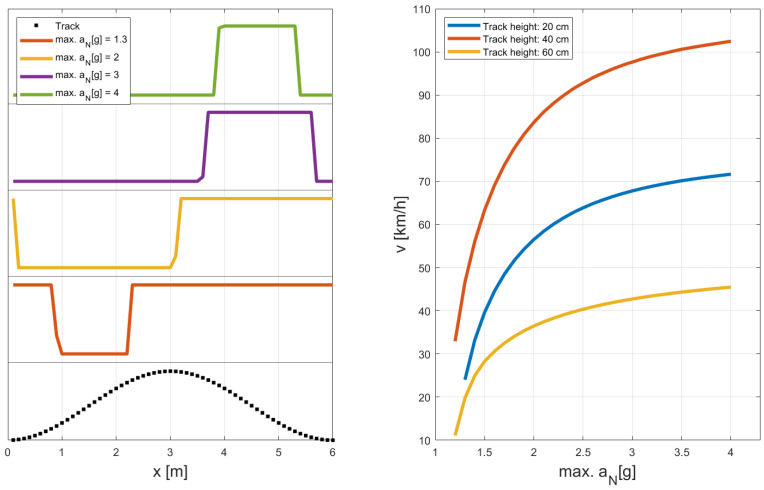
Variation of the maximal body force of the athlete on a track of length L= 6.0 m and a sinusoidal height profile with three different heights (0.20 m; 0.40 m; 0.60 m) between the lowest and the highest point: (**left**) Sinusoidal track profile with 0.20 m track height (dotted black line), dynamics of the body force as applied by athlete for different maximal body forces for the track height 0.2 m only. The graphics is scaled and shifted in vertical direction for clarity. (**right**) Variation of the athlete’s medium velocity in x-direction under variation of the maximal body force for three different track heights (0.20 m; 0.40 m; 0.60 m).

For the energy pumping on a track with sinusoidal height profile and circular conditions we find that there is a lower limit of athlete’s maximal body force of approx. (1.2–1.3) g, below that the NLP does not have a solution, and accordingly energy pumping cannot be performed. For maximal body forces above this limit, the velocity increases steadily as shown in Figure 5 (right). Also, we see a pronounced influence of the track height on the speed. For track heights lower than $\left(h^{max}-h^{min}\right)$, the velocity increases as the track height increases. In these cases the athlete is able to jump over the roller. But as the track height is larger than $\left(h^{max}-h^{min}\right)$, the velocity decreases as the athlete is not able anymore to jump over the rollers.

In Figure 5 (left), we present the optimal body force as applied by the athlete for 4 distinct values for energy pumping on a track with track height 0.20 m. We see that the quality of the optimal movement remains untouched: In all cases we have a switch between a flying phase and a force phase. And in all cases, the athlete applies his or her maximal body force, but for a high maximal body force, the flying phase can be long, and the force phase is short. On the other hand, for a small maximal body force, the athlete can only perform a movement with a short flying phase, and the force phase needs to be long. Clearly, the exact timing of the flying and the force phase is fundamental to achieve optimal energy pumping.

### 3.3. Qualitative Comparison of Optimal Movement Pattern with Data Derived from Video-Recorded BMX-Athlete

Finally, we present a qualitative comparison of (a) the results achieved for the optimal movement pattern of the COM, to (b) a single elite athlete’s movement pattern as taken from video from a public source [22]. This video does not allow for a quantitative comparison. The video only allows for a simple qualitative comparison. It is subject to future research to perform more precise and controlled measurements of movement patterns of a group of elite athletes for a more fundamental comparison.

**Figure 6:**
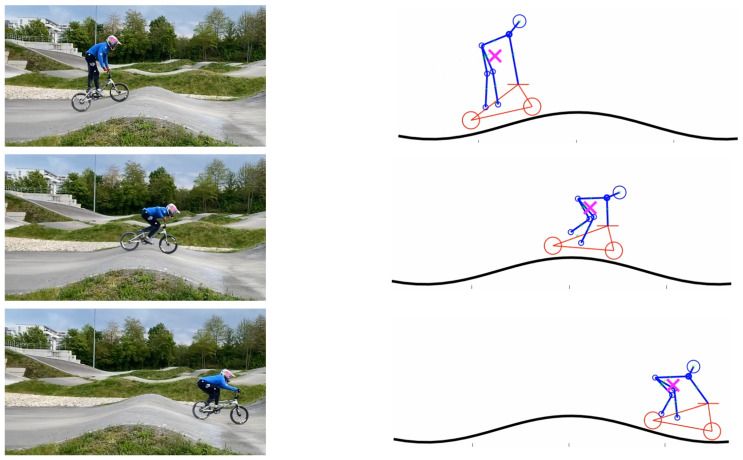
Extraction of COM’s movement pattern from a video recording of an BMX elite athlete during energy pumping. Left column: snapshots of athlete’s movement. Right column: Tagging of athlete’s body segments, the bike, and the computed COM (pink cross).

For this, we applied manual tagging of the athlete’s bike and athlete’s body segments (head, shoulder, arms, upper leg, lower leg) for every single picture of the video sequence (see Figure 6). Then, the COM of the athlete and the bike was computed on the basis of standard weight distributions for human bodies. With this, we were able to compute the movement of the COM in the 2-dimensional space and derive COM’s distance to the track.

**Figure 7:**
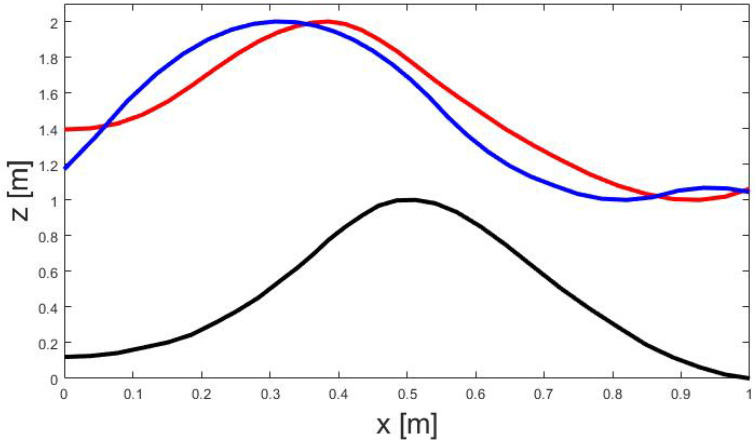
Movement patterns for energy pumping on a single bump (black line): movement of the COM as extracted from the video sequence (red line), and optimal movement pattern computed by solving the discrete optimal control problem.

As presented in Figure 7, the movement patterns are similar but not identical. They share common features: jump phase in the uphill section of the bump, and landing phase in the downhill section of the bump. The maximal stretching of the body is observed in both cases slightly before the maximum of the bump.

## 4. Discussion

### 4.1. General

We present our results as a first step towards a full understanding of optimal movement patterns for energy pumping. Clearly, there is a tremendous space for future research and important improvements. In our eyes, the following future research directions seem to be most promising, and interesting:

1. As the inverted pendulum model presented in this paper is highly simplified, we propose to increase the accuracy of the model by integrating more degrees of freedom into the model (modeling of athlete’s joints, modeling of athlete’s equipment). With this, the physical model of the athlete’s movement will be closer to reality, and therefore it is more likely that concise and accurate conclusions can be drawn from the computed optimal movement patterns to support athletes, and contribute to sport science. For instance, we expect results, which are closer to reality, if the athlete does not only have a single isolated contact point (such as in the inverted pendulum model), but instead has multiple contact points (as it is in biking), or a line of contact points (as it is skiing).
2. Perform high-quality measurements of movement patterns of athletes performing energy pumping in such different disciplines, such as BMX-racing, or ski-cross. On this basis, it would be highly interesting to be able to compare movement patterns of athletes from different disciplines to the results of the optimal movement patterns as presented in this paper.

### 4.2. Learnings for Training and Competition in Sports

From our study we can draw some conclusions for athletes and coaches engaged in sports in which energy pumping is an success factor (like BMX racing, or ski cross):

1. The results fully agree with athlete’s experience that (a) leg force should be applied before the bump to avoid uphill movement, and (b) in the landing or compression phase, leg force is needed for landing.
2. In any case, if facing high or low bumps, the athlete should use his or her maximal leg force, since the horizontal acceleration depends on the maximal leg force applied.
3. The correct and exact timing of jump and landing phase is crucial for the energy pumping. Obviously, as horizontal velocities increase it is increasingly difficult for the athlete to achieve the correct timing of the jumping and landing phase.
4. The optimal path can be used for evidence-based coaching.
5. As a result of our study, the major advice for athletes could be phrased in simple words as: Avoid riding uphill, it’s better to jump over the hill. Generally when driving downhill try to push your center of mass upwards.

### 4.3. Limitations

As this study is theoretical in nature, its focus lies on defining a proper dynamical model, defining a proper continuous and discrete optimal control problem, and solving the latter numerically according to the state-of-the-art. Therefore, it is important for us to highlight the limitations of our work:

1. In this study there was no quantitative validation. In the future a proper quantitative validation is required based on subject-specific scaling of the model and motion analysis on the field.
2. While the simple inverted-pendulum model turns out to be an adequate model to understand energy pumping in a generic way, it might be oversimplified to deliver a detailed description of the movement. As such, in the future, we expect more complex models will be required for a detailed description of optimal movement patterns in energy pumping.
3. As the inverted-pendulum model possesses a single contact point between the athlete and the ground, it might be oversimplified for a detailed description of cycling (with 2 contact points), and skiing (with a extended line of contact). Therefore, in the future, to describe the optimal movement patterns in those sports in detail an extension of the inverted-pendulum model is required.

## 5. Conclusions

The inverted pendulum model, which is already highly simplified, in combination with the numerical solution of an optimal control problem turn out to be valuable tools to increase the fundamental understanding of the effect of energy pumping. Although, more work has to be done to increase the accuracy of the model on one hand, and on the other hand to provide a more exact and broader basis of experimental measurements, our research allows already for practically relevant conclusions, and even more importantly, provides a guide to the eye for the future research. The authors find it especially promising to investigate in the future optimal movement patterns in other sports like gymnastics, skiing, or track and field.

## Data Availability

Not applicable.
